# Real-World Efficacy and Safety of Zishenyizhi Pill for Cognitive Impairment Associated With Cerebral Small Vessel Disease: Protocol for a Multicenter Prospective Observational Study

**DOI:** 10.2196/77661

**Published:** 2025-12-08

**Authors:** Xiongxing Sun, Yao Xie, Rui Fang, Shanshan Zeng, Shigao Lin, Xukun Tang, Jie Tang, Tenghui Hu, Yuhui Zhang, Le Xie, Dahua Wu

**Affiliations:** 1 Graduate School of Hunan University of Chinese Medicine Changsha China; 2 Hunan Provincial Hospital of Integrated Traditional Chinese and Western Medicine (The Affiliated Hospital of Hunan Academy of Traditional Chinese Medicine) Changsha China; 3 Institute of Clinical Pharmacology Hunan Academy of Chinese Medicine Changsha China

**Keywords:** cerebral small vessel disease, cognitive impairment, zishenyizhi pill, real-world studies, traditional Chinese medicine

## Abstract

**Background:**

Cerebral small vessel disease (CSVD) is a leading cause of vascular cognitive impairment, contributing to 36% to 67% of vascular dementia cases. Current therapies, primarily cholinesterase inhibitors, offer limited efficacy and significant adverse effects. Traditional Chinese medicine, particularly the zishenyizhi pill, which is a multiherb formulation targeting yin deficiency and blood stasis, has shown promise in preliminary studies but lacks robust clinical validation.

**Objective:**

We designed this study to evaluate the real-world efficacy and safety of zishenyizhi pill combined with conventional Western therapy for CSVD-related cognitive impairment.

**Methods:**

This multicenter, prospective, nonrandomized controlled trial enrolled 246 patients from 4 institutions in Hunan province, China. Participants were stratified into 2 groups: the intervention group received zishenyizhi pill plus standard Western therapy, whereas the control group received conventional integrated Chinese-Western therapy. Cognitive outcomes (Montreal Cognitive Assessment, Mini-Mental State Examination, Vascular Dementia Assessment Scale cognitive subscale, and Trail Making Test parts A and B), traditional Chinese medicine syndrome scores, and safety parameters were assessed over 12 weeks.

**Results:**

Participant recruitment is scheduled to commence in April 2025, with an anticipated enrollment of 246 participants by December 2026. At present, patient recruitment is underway, and preliminary analysis of medical record data is being conducted. Final data collection is expected to conclude by April 2027, with results projected for publication in September 2027.

**Conclusions:**

The anticipated findings suggest that zishenyizhi pill may improve multiple cognitive domains in patients with cognitive impairment associated with CSVD while demonstrating a favorable safety profile. If validated, zishenyizhi pill could serve as a safe and effective adjunctive therapy for CSVD-related cognitive dysfunction.

**Trial Registration:**

International Traditional Medicine Clinical Trial Registry ITMCTR2025000553; https://itmctr.ccebtcm.org.cn/mgt/project/view/-4790836881284495026

**International Registered Report Identifier (IRRID):**

DERR1-10.2196/77661

## Introduction

Cerebral small vessel disease (CSVD) is a clinical, imaging, and pathological syndrome caused by diverse etiologies affecting cerebral small arteries, arterioles, capillaries, venules, and small veins. Its primary neuroimaging manifestations include recent small subcortical infarcts, perivascular spaces, presumed vascular origin lacunes, white matter hyperintensities, cerebral microbleeds, and cerebral atrophy [1,2]. While patients with CSVD may present with acute-onset symptoms such as lacunar infarcts or hemorrhagic stroke, most exhibit insidious progression characterized by gradual cognitive decline, gait disturbances, affective disorders, and bladder or bowel dysfunction [3,4]. During the acute phase of CSVD evolution, pathological changes predominantly manifest as lacunar infarcts and intraparenchymal hemorrhage, accounting for approximately one-fifth of symptomatic stroke cases [5,6]. In chronic stages, the disease progresses to hemodynamically significant cerebral hypoperfusion, where persistent blood flow dysregulation induces multidimensional neural damage [7,8]. This pathophysiological cascade clinically correlates with progressive cognitive deterioration (encompassing memory, executive function, attention, language, and visuospatial domains), motor coordination deficits, autonomic dysfunction, and neuropsychiatric syndromes. Notably, CSVD represents one of the most prevalent etiologies of cognitive impairment, typically exhibiting a characteristic decline pattern marked by predominant deficits in attention, processing speed, and executive function, with relatively preserved episodic memory [9]. Epidemiological studies indicate that CSVD-related cognitive dysfunction contributes to 36% to 67% of vascular dementia (VaD) cases [10], often leading to severe functional disability, increased mortality, and substantial socioeconomic burdens.

The underlying mechanisms of cognitive impairment in CSVD remain incompletely elucidated. Neuroimaging modalities, particularly magnetic resonance imaging, have provided critical insights into potential pathophysiological correlates. Current evidence demonstrates associations among lacunar infarcts, white matter hyperintensities, cerebral atrophy, and cognitive deficits [11-13], whereas the presence and burden of cerebral microbleeds exhibit modest yet significant impacts on executive function and information processing speed [14,15]. However, the role of perivascular spaces in cognitive decline remains inconclusive based on extant data [16].

Despite the high prevalence and substantial societal burden of CSVD-related cognitive impairment, targeted therapeutic research remains limited. Current interventions predominantly derive from VaD studies, where cholinesterase inhibitors (eg, donepezil, rivastigmine, and galantamine) are used to enhance synaptic acetylcholine levels and mitigate amyloid β protein generation and deposition, thereby ameliorating cognitive dysfunction [17-21]. While these agents demonstrate modest cognitive benefits, they are associated with clinically significant adverse effects, including severe gastrointestinal disturbances (eg, nausea and vomiting), dizziness, and bradycardia [22,23]. Other pharmacotherapies with putative efficacy in vascular cognitive impairment (VCI), such as *Ginkgo biloba* extracts and nimodipine, require further validation through randomized controlled trials (RCTs) [24,25]. Traditional Chinese medicine (TCM) offers a unique therapeutic paradigm for CSVD. The yin deficiency and blood stasis theory constitute the principal TCM pathogenesis framework, positing dual involvement of the kidney and brain. Guided by this theory, the zishenyizhi decoction (nourishing the kidney and enhancing intelligence decoction) has been empirically used to modulate CSVD progression.

Preliminary clinical studies have demonstrated that zishenyizhi decoction significantly improves cognitive dysfunction in patients with CSVD, as evidenced by enhanced scores on the Mini-Mental State Examination (MMSE), Instrumental Activities of Daily Living Scale, National Institutes of Health Stroke Scale, and TCM syndrome evaluation metrics. Preclinical investigations using rodent models of chronic cerebral hypoperfusion further revealed that this formulation ameliorates ischemia-hypoxia–induced pathological changes, enhances spatial learning and memory performance, and reduces hippocampal damage. This prescription has been transformed into the Chinese patent medicine zishenyizhi pill and is widely used in clinical practice due to its clinical efficacy. The zishenyizhi pill is composed of 11 herbs, with the composition and functions shown in Table 1. However, the precise molecular mechanisms underlying its neuroprotective and cognitive-enhancing properties in CSVD are yet to be fully elucidated.

Real-world studies originating from pragmatic clinical trials within the domain of pharmacoepidemiology involve large-scale, long-term evaluations of therapeutic interventions under routine clinical practice conditions. These studies prioritize clinically meaningful outcome measures to assess both efficacy and safety, with a primary emphasis on external validity [26]. In contrast, RCTs focus on internal validity by investigating highly selected populations under strictly controlled experimental settings [27,28]. RCTs are conducted under the premise that findings derived from selected study populations can be generalized to broader target populations. This study design is widely regarded as the gold standard for generating high-reliability evidence, with most clinical guidelines formulating recommendations based on outcomes extrapolated from RCT data [29]. However, a critical limitation stems from the stringent inclusion and exclusion criteria, which may result in study cohorts that inadequately reflect real-world clinical populations and compromise generalizability to broader patient groups. Such restricted participant selection introduces selection bias [30,31]. Due to the particularity and complexity of its theoretical system, through preliminary exploration, we believe that real-world studies are more suitable for the field of TCM. Therefore, we will conduct a real-world study on the treatment of cognitive impairment in CSVD (syndrome of yin deficiency and blood stasis) with zishenyizhi pill. The aim is to clarify the clinical efficacy and medication characteristics of zishenyizhi pill in treating patients with cognitive impairment due to CSVD and provide evidence-based support for its use.

**Table 1:** Composition, effects, and pharmaceutical action of the Chinese herbal medicines that constitute zishenyizhi pill.

Chinese name	Latin name	Plant part	Effect (TCM^a^)	Pharmaceutical action	Dose (g)
Heshouwu	*Pleuropterus multiflorus*	Fruit	Nourishes essence and blood	Antioxidation, hypolipidemia regulation, and hepatoprotection	5
Gouqizi	*Lycii fructus*	Fruit	Nourishes the liver and kidney	Immunoregulation, anti-inflammation, and liver protection	5
Sangshen	*Mori fructus*	Fruit	Nourishes yin and blood and promotes fluid	Immunoregulation and antioxidation	5
Yinxingye	*Ginkgo biloba*	Leaf	Promotes blood circulation for removing blood stasis and obstruction in collaterals	Antioxidation, neuroprotection, and anti-inflammation	5
Danshen	*Salviae miltiorrhizae*	Root	Promotes blood circulation for removing blood stasis and obstruction in collaterals	Neuroprotection, antioxidation, and improvement of microcirculation	6
Yizhiren	*Alpinia oxyphylla*	Fruit	Nourishes the spleen and kidney	Neuroprotection, memory enhancement, and anti-inflammation	5
Shichangpu	*Acorus tatarinowi*	Tuber	Harmonizes the stomach, resolves phlegm, and opens the orifices	Neuroprotection, anticonvulsion, and antioxidation	5
Yujin	*Curcuma aromatica*	Tuber	Promotes qi circulation, activates blood flow, and alleviates pain	Anticancer, anti-inflammation, and antioxidation	5
Yuanzhi	*Polygala tenuifolia Willd.*	Root	Sedates the heart to stabilize the spirit and resolves phlegm to restore consciousness	Neuroprotection, antidepression, anti-inflammation, and antioxidation	5
Quanxie	Karsch *Olivierus martensii*	Dry body	Dispels pathogenic wind for resolving convulsion	Analgesia, anti-inflammation, and neuroprotection	1
Shanzha	*Crataegi fructus*	Fruits	Harmonizes the stomach and aids digestion	Neuroprotection	5

^a^TCM: traditional Chinese medicine.

## Methods

### Study Setting

**Figure 1:**
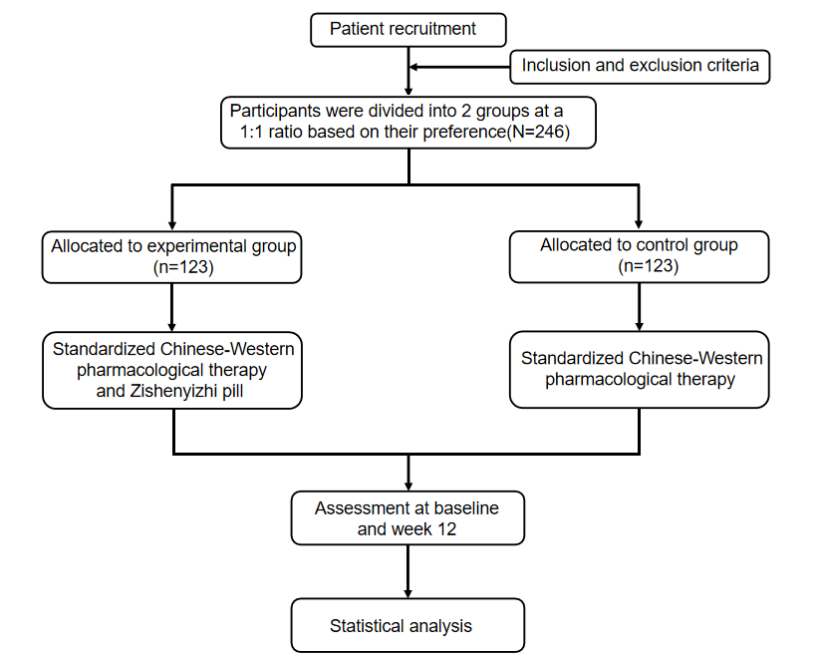
Flowchart of the study.

This multicenter prospective observational nonrandomized controlled study (registered at the International Traditional Medicine Clinical Trial Registry; ITMCTR2025000553) enrolled 246 patients with CSVD-associated cognitive impairment of yin deficiency and blood stasis syndrome from 4 institutions—Hunan Provincial Hospital of Integrated Traditional Chinese and Western Medicine, Yiyang First Hospital of Traditional Chinese Medicine, Liuyang Hospital of Traditional Chinese Medicine, and Shimen County Hospital of Traditional Chinese Medicine. Eligible participants were stratified into 2 groups based on zishenyizhi pill use: the intervention group received guideline-based conventional Western medical therapy combined with zishenyizhi pill for 12 weeks, whereas the control group received guideline-based conventional integrated Chinese-Western therapy. Treatment for all enrolled participants was initiated during the stable phase of their outpatient visit or inpatient stay, immediately following the baseline assessment and confirmation of eligibility. Over the 12-week study period, multidomain cognitive assessments (executive function, attention, and processing speed) were conducted to evaluate the efficacy of zishenyizhi pill in CSVD-related cognitive impairment and explore its underlying mechanisms. The trial workflow is summarized in Figure 1. The evaluations and visits will be conducted according to the testing schedule in Table 2.

**Table 2:** Study timeline.

Assessment		Baseline	Midpoints		End point
		0 wk	4 wk	8 wk	12 wk
Informed consent		✓			
Inclusion criteria		✓			
Exclusion criteria		✓			
General information		✓			
Medical history		✓			
TCM^a^ syndrome criteria		✓			✓
**Primary outcome measure**					
	MoCA^b^	✓			✓
**Secondary outcome measures**					
	VaDAS-cog^c^	✓	✓	✓	✓
	MMSE^d^	✓	✓	✓	✓
	TMT-A^e^	✓	✓	✓	✓
	TMT-B^f^	✓	✓	✓	✓
	BNT-2^g^	✓	✓	✓	✓
	HVLT^h^	✓	✓	✓	✓
	CDT^i^	✓	✓	✓	✓
	IADL^j^	✓	✓	✓	✓
	EQ-5D-5L	✓	✓	✓	✓
	NPI^k^	✓	✓	✓	✓
	HADS^l^	✓	✓	✓	✓
	TCM syndrome criteria	✓	✓	✓	✓
**Safety assessments**					
	Blood, urine, and stool routine test	✓	✓	✓	✓
	Liver and kidney function	✓	✓	✓	✓
	Coagulation function	✓	✓	✓	✓
	Electrolytes	✓	✓	✓	✓
	Cardiac enzymes	✓	✓	✓	✓
	Lipid profile	✓	✓	✓	✓
	Blood glucose	✓	✓	✓	✓
	ECG^m^	✓	✓	✓	✓
	Efficacy evaluation		✓	✓	✓

^a^TCM: traditional Chinese medicine.

^b^MoCA: Montreal Cognitive Assessment.

^c^VaDAS-cog: Vascular Dementia Assessment Scale cognitive subscale.

^d^MMSE: Mini-Mental State Examination.

^e^TMT-A: Trail Making Test part A.

^f^TMT-B: Trail Making Test part B.

^g^BNT-2: Boston Naming Test–Second Edition.

^h^HVLT: Hopkins Verbal Learning Test.

^i^CDT: clock drawing test.

^j^IADL: Instrumental Activities of Daily Living Scale.

^k^NPI: Neuropsychiatric Inventory.

^l^HADS: Hospital Anxiety and Depression Scale.

^m^ECG: electrocardiogram.

### Training of Assessors and Quality Control

To ensure the consistency and reliability of the cognitive assessments, all neuropsychological evaluators across the 4 centers underwent centralized, standardized training before study initiation. Training sessions covered detailed administration protocols, scoring criteria, and interpretation guidelines for each cognitive tool (eg, Montreal Cognitive Assessment [MoCA], MMSE, Vascular Dementia Assessment Scale cognitive subscale [VaDAS-cog], and Trail Making Test parts A [TMT-A] and B [TMT-B]). Calibration exercises were conducted to minimize interrater variability, and periodic quality checks were implemented throughout the study.

### Participants

This study focuses on patients with cognitive impairment due to CSVD with TCM syndrome of yin deficiency and blood stasis treated in the outpatient and inpatient departments of 4 institutions: Hunan Provincial Hospital of Integrated Traditional Chinese and Western Medicine (affiliated hospital of Hunan Academy of Chinese Medicine), Yiyang First Hospital of Traditional Chinese Medicine, Liuyang Hospital of Traditional Chinese Medicine, and Shimen County Hospital of Traditional Chinese Medicine.

### Inclusion Criteria

The inclusion criteria are as follows: (1) Western medicine diagnosis meeting the diagnostic criteria for CSVD-associated cognitive impairment, (2) TCM syndrome meeting the yin deficiency and blood stasis syndrome criteria (the Dementia Syndrome Differentiation and Classification Scale is used for syndrome judgment, and the syndrome is established when the score is ≥7; the rating details are shown in Multimedia Appendix 1), (3) age ≥60 years, and (4) agreement from the patients or their family members and signature of an informed consent form.

### Exclusion Criteria

The exclusion criteria are as follows: (1) coexisting neurological disorders causing cognitive impairment, including Alzheimer disease, frontotemporal dementia, Lewy body dementia, Parkinson disease, multiple sclerosis, traumatic brain injury, autoimmune disorders, or genetic diseases; (2) impaired consciousness, unstable vital signs (eg, blood pressure, respiratory rate, or heart rate), or life-threatening conditions (such as severe cardiac, hepatic, renal, or endocrine disorders); (3) inability to comprehend or comply with study procedures or follow-up due to psychiatric illnesses, cognitive or emotional disorders, or severe visual or auditory impairments; (4) severe hepatic or renal dysfunction (alanine transaminase or aspartate transaminase of >2 times the upper normal limit; creatinine of >1.5 times the upper normal limit); (5) previous use of kidney-nourishing or blood-activating herbal therapies within 2 weeks before enrollment; and (6) hypersensitivity to any component of the zishenyizhi pill.

### Recruitment of Participants

All participants will be recruited through posters from inpatients and outpatients of 4 institutions: Hunan Provincial Hospital of Integrated Traditional Chinese and Western Medicine (affiliated hospital of Hunan Academy of Chinese Medicine), Yiyang First Hospital of Traditional Chinese Medicine, Liuyang Hospital of Traditional Chinese Medicine, and Shimen County Hospital of Traditional Chinese Medicine.

### Interventions

All participants were stratified into 2 groups based on zishenyizhi pill administration: the intervention group and the control group, each comprising 123 patients. Both groups received standardized Western pharmacological therapy, including antihypertensive treatment, antiplatelet therapy, and lipid-lowering therapy. The intervention group additionally received the zishenyizhi pill at a dosage of twice daily for 12 consecutive weeks.

### Outcomes

#### Primary Outcome

The MoCA assessed the degree of global cognitive function at baseline and 12 weeks after the intervention.

#### Secondary Outcomes

The VaDAS-cog assessed the degree of attention, executive function, and related cognitive domains at baseline and 12 weeks after the intervention. The MMSE assessed the degree of orientation, memory, and calculation ability at baseline and 12 weeks after the intervention. The TMT-A assessed the degree of visuospatial ability, psychomotor speed, and visual attention at baseline and 12 weeks after the intervention. The TMT-B assessed the degree of executive function, processing speed, and cognitive flexibility at baseline and 12 weeks after the intervention. The Boston Naming Test–Second Edition assessed the degree of language function (naming ability) at baseline and 12 weeks after the intervention. The Hopkins Verbal Learning Test assessed the degree of memory function (verbal memory) at baseline and 12 weeks after the intervention. The clock drawing test assessed the degree of visuospatial skills and executive function at baseline and 12 weeks after the intervention. The Instrumental Activities of Daily Living Scale assessed the degree of functional independence in daily living at baseline and 12 weeks after the intervention. The EQ-5D-5L assessed health-related quality of life at baseline and 12 weeks after the intervention. The Neuropsychiatric Inventory assessed the severity of neuropsychiatric symptoms at baseline and 12 weeks after the intervention. The Hospital Anxiety and Depression Scale assessed the severity of anxiety and depressive symptoms at baseline and 12 weeks after the intervention. The TCM syndrome criteria assessed the severity of TCM syndromes at baseline and 12 weeks after the intervention.

### Safety Evaluation and Adverse Events

Safety assessments included electrocardiogram, blood routine tests, liver function tests, renal function tests, cardiac enzyme level, blood glucose level, lipid profile, urine test, and stool routine test. Adverse events were comprehensively documented in case report forms (CRFs) throughout the study period. Serious adverse events—defined as those resulting in disability, operational incapacity, life-threatening conditions, or death—required immediate notification to both the investigators and the ethics committee. The research team provided clinical guidance and therapeutic interventions to affected participants while determining their eligibility for continued study participation.

### Sample Size

The primary objective of this trial was to evaluate the therapeutic efficacy of zishenyizhi pill in patients with CSVD-related cognitive impairment. The sample size was estimated based on an anticipated superiority in the MoCA score among the intervention group compared to the control group. On the basis of previous pilot data and clinical judgment, we assumed a mean difference (δ) in MoCA score change of 0.5 between the 2 groups, with a common SD (σ) of 1.52. A 2–independent-sample *t* test was used for the calculation. With a significance level (α; 2-sided) set at .05, a desired power (1 – β) of 90% for detecting this difference, and an allocation ratio (*K*) of 1:1, the required sample size per group was calculated using the following formula for a superiority trial: 
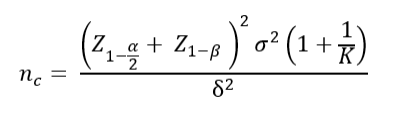
 . As a result, 98 participants were needed in the experimental and control groups. Accounting for a 20% anticipated dropout rate, we planned to enroll 123 participants per group, totaling 246.

### Combination Medications

Throughout the trial duration, all concomitant medications will undergo comprehensive monitoring. Additional use of TCM formulations with kidney-nourishing and blood-activating properties is strictly prohibited to ensure protocol compliance and minimize confounding effects.

### Statistical Analysis

All statistical analyses will be conducted using the SPSS software (version 25.0; IBM Corp). The primary analysis will follow the intention-to-treat principle. Measurement data that adhere to a normal distribution will be expressed as means and SDs and compared between groups using the independent-sample *t* test. For intragroup comparisons (eg, baseline vs 12-week follow-up), paired *t* tests will be used. Data that do not follow a normal distribution will be expressed as medians and ranges, and the Mann-Whitney *U* test will be used for intergroup comparisons. Count data and categorical variables will be expressed as numbers and percentages and compared using the Pearson chi-square test or Fisher exact test as appropriate.

For the primary outcome analysis, the change in MoCA score from baseline to 12 weeks will be compared between groups using an analysis of covariance adjusting for the baseline MoCA score to enhance precision. A similar analysis of covariance approach will be applied to analyze changes in secondary continuous outcomes (eg, MMSE, VaDAS-cog, and TMT-A and TMT-B scores), with respective baseline scores included as covariates. To explore independent factors associated with cognitive improvement, significant variables identified in univariate analyses will be included in a multivariate linear regression model. All tests will be 2-tailed, and a *P* value of <.05 will be considered statistically significant.

### Data Management and Quality Control

Data will be primarily collected using paper-based CRFs. To ensure data quality and accuracy, all data from the CRFs will be independently entered into a designated electronic database by 2 researchers. This process of double data entry and cross-verification will help minimize input errors. In case of inconsistent entries or identified omissions, the original CRFs will be rechecked for clarification and correction. To protect participant confidentiality, all personal identifiers will be removed and replaced with unique coded identification numbers in all electronic databases and analysis reports. The original paper CRFs will be securely stored in locked file cabinets at the principal investigator’s institution. The electronic database will be stored on a password-protected computer, with access restricted to authorized research personnel only. Participant personal information will not be disclosed under any circumstances without their written consent. After the database is locked, no further alterations will be permitted.

### Ethical Considerations

This study was approved by the Medical Ethics Committee of the Hunan Academy of Traditional Chinese Medicine Affiliated Hospital ({2025}29). This study will be conducted in accordance with the ethical principles of the Declaration of Helsinki. Informed consent will be obtained from all participants or their legal guardians before their enrollment in the study (informed consent is displayed in Multimedia Appendix 2). To protect participant privacy, all personal identifiers will be removed and replaced with a unique code; the list linking codes to identities will be stored separately under strict security measures.

## Results

This study was funded by the National Natural Science Foundation of China General Program (grant 8237153821). Participant recruitment commenced in April 2025 across the 4 participating centers and is projected to be completed by December 2026. Final data collection, including all 12-week follow-up assessments, is anticipated to conclude by April 2027. As of November 2025, a total of 47 participants have been enrolled, 28 of whom have completed the follow-up assessments. Data analysis will commence following the completion of data collection. The primary results of this study are expected to be submitted for publication in September 2027.

## Discussion

### Rationale and Context

VCI is a recently recognized clinical entity primarily caused by cerebrovascular diseases [32,33]. Diverse etiologies contribute to VCI, including CSVD, large artery atherosclerosis, intracerebral hemorrhage, cardioembolism, and other less common stroke mechanisms [34,35]. VCI encompasses a spectrum of cognitive deficits, ranging from mild cognitive impairment to VaD, which arises from ischemic or hemorrhagic vascular pathologies either independently or synergistically with neurodegenerative processes such as Alzheimer disease [36]. Epidemiological projections estimate that the global burden of VCI will escalate to 100 million cases by 2050, incurring economic losses approaching US $4 trillion [37]. Although the current prevalence of VCI remains relatively low in low- and middle-income countries undergoing early demographic transitions, the most rapid growth rates in incidence are occurring there, suggesting a significant future disease burden [38]. CSVD represents a critical subtype of VCI and is the most prevalent etiological contributor to VaD in clinical practice [39]. Despite the high incidence and substantial societal burden of CSVD-related cognitive impairment, targeted therapeutic research remains scarce. Current interventions predominantly focus on VaD populations, with limited studies addressing subcortical VCI. However, TCM has long been associated with dementia treatment, supported by extensive research on TCM-based interventions [40]. TCM offers distinct advantages, including fewer adverse effects, cost-effectiveness, suitability for long-term use, and preventive potential [41], positioning it as a significant complementary and alternative therapeutic option for CSVD-related cognitive dysfunction.

The zishenyizhi pill, an empirical formula rooted in TCM theory, targets the core pathogenesis of CSVD by addressing yin deficiency and blood stasis. Preliminary clinical observations suggest its safety and efficacy in managing CSVD-related cognitive impairment; however, there is a lack of robust evidence from high-quality studies. To rigorously evaluate the zishenyizhi pill’s therapeutic impact, this study uses the MoCA as the primary end point due to its superior sensitivity and specificity in detecting cognitive deficits compared to the MMSE. Secondary outcomes will assess multidimensional cognitive domains and functional efficacy through validated neuropsychological scales.

### Limitations

Nevertheless, this study is subject to several methodological limitations. The nonrandomized observational design carries an inherent risk of selection bias and confounding. Despite stratification based on zishenyizhi pill use, baseline differences in patient characteristics—such as disease severity, comorbidities, and socioeconomic status—may persist between the intervention and control groups, potentially influencing outcome interpretation. The absence of randomization compromises internal validity as unmeasured confounders, including genetic predispositions and lifestyle factors, could independently affect cognitive trajectories.

Although real-world studies prioritize external validity, the limited control over confounding variables constrains the ability to establish causal inferences regarding the therapeutic efficacy of zishenyizhi. In addition, the sample size, while feasible for this preliminary investigation, was not derived from a formal superiority test calculation and was based on a conservative effect size estimate. Consequently, this study may be underpowered to detect differences smaller than 0.5 points on the MoCA, which could represent clinically meaningful but more modest treatment effects, particularly for secondary outcomes. This also limits the potential for meaningful subgroup analyses to explore personalized treatment responses.

Furthermore, the reliance on neuropsychological scales as primary end points without incorporating neuroimaging biomarkers such as magnetic resonance imaging–based quantification of white matter hyperintensity volume or microbleed burden restricts mechanistic interpretation. Objective biomarkers are essential to determine whether cognitive improvements correspond to structural or molecular changes in CSVD pathology. Finally, the 12-week observation period may be insufficient to evaluate the long-term efficacy, safety, and sustained cognitive benefits of zishenyizhi given the chronic and progressive nature of CSVD. Short-term assessment could miss delayed treatment effects, disease progression patterns, or late-emerging adverse events, which are critical in the context of vascular cognitive disorders.

### Future Directions

Despite these constraints, this study represents a pivotal step in bridging TCM paradigms with modern clinical research methodologies. The limitations identified herein provide a clear road map for future research. To definitively establish the efficacy of zishenyizhi, subsequent studies should prioritize a multicenter RCT design to overcome the selection bias inherent in this observational approach. This RCT should be explicitly powered for superiority testing, with a sample size calculated based on a formally justified effect size for a prespecified primary cognitive end point. To elucidate the neurobiological mechanisms and move beyond reliance on neuropsychological scales alone, future trials should incorporate multimodal neuroimaging and fluid biomarkers. Furthermore, given the chronic progression of CSVD, extended intervention and follow-up periods are essential to evaluate the long-term sustainability of cognitive benefits and the intervention’s impact on disease progression. Finally, leveraging larger cohorts will enable subtype stratification based on clinical, imaging, or biomarker profiles to identify patient subgroups most likely to benefit from zishenyizhi, thereby advancing toward personalized medicine in CSVD. These concerted efforts will be crucial in robustly validating this TCM intervention and advancing its integration into global therapeutic strategies for CSVD.

### Conclusions

This ongoing multicenter real-world study represents a critical effort to evaluate the integrative use of zishenyizhi pill with conventional therapy for cognitive impairment in CSVD. By combining TCM principles with modern clinical methodologies, this trial aims to address the unmet need for targeted therapies in CSVD-related cognitive decline. While the nonrandomized design and reliance on neuropsychological end points may limit causal inferences, this study’s pragmatic approach provides valuable insights into TCM’s applicability in real-world settings. Pending completion, the findings will lay the groundwork for future randomized trials to validate efficacy, elucidate mechanisms (eg, through neuroimaging biomarkers), and optimize personalized treatment strategies. This protocol highlights the potential of TCM as a complementary paradigm in global dementia research, bridging traditional knowledge with evidence-based practice.

## Appendix

Multimedia Appendix 1 Dementia Syndrome Differentiation and Classification Scale.

Multimedia Appendix 2 Informed consent form.

## Abbreviations

CRF: case report form
CSVD: cerebral small vessel disease
MMSE: Mini-Mental State Examination
MoCA: Montreal Cognitive Assessment
RCT: randomized controlled trial
TCM: traditional Chinese medicine
TMT-A: Trail Making Test part A
TMT-B: Trail Making Test part B
VaD: vascular dementia
VaDAS-cog: Vascular Dementia Assessment Scale cognitive subscale
VCI: vascular cognitive impairment

## Data Availability

Currently, no data have been generated. Relevant data will be available from the corresponding author on reasonable request.
