# Supplementary material for: Real-World Efficacy and Safety of Zishenyizhi Pill for Cognitive Impairment Associated With Cerebral Small Vessel Disease: Protocol for a Multicenter Prospective Observational Study
Source: JMIR Res Protoc. 2025 Dec 8;14:e77661. doi: 10.2196/77661 (PMC12723357; doi:10.2196/77661)
Supplement: Multimedia Appendix 1 [file resprot_v14i1e77661_app1.docx]

Dementia Syndrome Differentiation and Classification Scale

| **TCM syndrome** | **Acute ischemic stroke–related symptoms and signs** | **Score** |
| --- | --- | --- |
|  | Bowel and Urinary Incontinence | 5 |
|  | Urinary Incontinence | 4 |
|  | Frequent Nocturia | 4 |
| **Kidney Deficiency** | Soreness and Weakness of the Lower Back and Knees | 4 |
|  | Decreased Libido | 3 |
|  | Tinnitus and Hearing Loss | 2 |
|  | Deep and Weak Chi Pulse | 1 |
|  | Loose and Unformed Stools | 5 |
|  | Poor Appetite with Reduced Food Intake | 4 |
|  | Postprandial Abdominal Distension | 4 |
| **Spleen Deficiency** | Excessive Drooling | 3 |
|  | General Fatigue and Lethargy | 3 |
|  | Pale Tongue with Teeth Marks | 3 |
|  | Slow Pulse | 1 |
|  | Shortness of Breath with Reluctance to Speak | 5 |
|  | Profuse Spontaneous Sweating | 4 |
|  | Mental Fatigue and Physical Lassitude | 4 |
| **Qi Deficiency** | Pale and Lusterless Complexion | 3 |
|  | Easily Frightened with Timidity | 3 |
|  | Pale Tongue Body | 2 |
|  | Weak Pulse | 2 |
|  | Pale Lips and Eyelids | 5 |
|  | Dull and Sallow Complexion | 4 |
|  | Dizziness with Blurred Vision | 4 |

| **Blood Deficiency** | Palpitations with Anxiety | 4 |
| --- | --- | --- |
|  | Insomnia with Excessive Dreaming | 3 |
|  | Numbness in Hands and Feet | 2 |
|  | Thready Pulse | 1 |
|  | Red Tongue with Scanty Moisture | 5 |
|  | Dry and Irritated Eyes | 4 |
|  | Emaciation or Progressive Weight Loss | 4 |
| **Yin Deficiency** | Dry and Hard Stools | 3 |
|  | Night Sweats | 3 |
|  | Scanty or Absent Tongue Coating | 3 |
|  | Thready and Rapid Pulse | 1 |
|  | Cold Limbs | 5 |
|  | Watery Diarrhea with Undigested Food | 5 |
|  | Chills with Huddling Posture | 3 |
| **Yang Deficiency** | Oliguria with Edema | 3 |
|  | Clear and Copious Urine | 3 |
|  | Pale Tongue with Slippery Coating | 3 |
|  | Deep and Weak Pulse | 1 |
|  | Soreness and Aching in Limbs and Legs | 5 |
|  | Dry and Brittle Teeth with Scorched Hair | 4 |
|  | Clumsy Movements | 4 |
| **Marrow Depletion** | Fatigue with Excessive Sleepiness | 4 |
|  | Persistent Dizziness | 3 |
|  | Small and Thin Tongue Body | 2 |
|  | Deep and Thready Pulse | 1 |
|  | Irritability and Proneness to Anger | 5 |
|  | Hallucinations and Delusions | 4 |
|  | Restlessness and Agitation | 4 |
| **Fire Disturbance** | Dizziness with Blurred Vision | 3 |
|  | Temporal and Vertex Headaches | 3 |
|  | Tinnitus Resembling Ocean Waves | 3 |
|  | Wiry Pulse | 1 |
|  | Mania with Restlessness | 5 |
|  | Delirium with Incoherent Speech | 4 |
|  | Abnormal or Inappropriate Behavior | 4 |
| **Toxic Excess** | Clouded Consciousness | 4 |
|  | Foul Breath with Constipation | 3 |
|  | Flushed Face with Red Eyes | 2 |
|  | Deep Red or Crimson Tongue | 1 |
|  | Expectoration of Thick Sputum and Saliva | 5 |
|  | Emotional Apathy | 4 |
|  | Greasy and Turbid Tongue Coating | 4 |
| **Phlegm Turbidity** | Obesity with Heavy Sensation in the Body | 3 |
|  | Poor Appetite with Nausea | 3 |
|  | Lethargy with Excessive Sleepiness | 2 |
|  | Slippery Pulse | 2 |
|  | Dark Tongue with Ecchymotic Spots | 5 |
|  | Pain Aggravated at Night | 4 |
|  | Fixed and Localized Pain | 4 |
| **Blood Stasis** | Dusky and Sallow Complexion | 4 |
|  | Cyanotic Lips and Nails | 3 |
|  | Bizarre Delusions or Irrational Thoughts | 2 |
|  | Choppy Pulse or Absent Pulse | 1 |
| Each syndrome is established when the score is ≥**7** (the sum of the highest scores of the respective diagnostic items). | | |
