# Supplementary material for: Real-World Efficacy and Safety of Zishenyizhi Pill for Cognitive Impairment Associated With Cerebral Small Vessel Disease: Protocol for a Multicenter Prospective Observational Study
Source: JMIR Res Protoc. 2025 Dec 8;14:e77661. doi: 10.2196/77661 (PMC12723357; doi:10.2196/77661)
Supplement: Multimedia Appendix 2 [file resprot_v14i1e77661_app2.docx]

Dear Madam/Sir:

Informed Consent Form

You are invited to participate in the "Real-world Study of Zishen Yizhi Pills in the Treatment of Cognitive Impairment Caused by Cerebral Small Vessel Disease (Yin Deficiency and Blood Stasis Syndrome)". Before you decide whether to participate in this study, please carefully read the following content, which will help you understand the purpose, process, and duration of this study, as well as the potential benefits, risks, and discomforts you may experience after participating. You can discuss it with your relatives and friends to help you make a decision. If you have any questions, please ask your research doctor, who will provide you with a full explanation.

# I. Research Background and Purpose

Cerebral small vessel disease (CSVD) is a series of clinical, imaging, and pathological syndromes caused by various etiologies affecting small arteries, arterioles, capillaries, venules, and small veins in the brain. The main imaging manifestations include recent subcortical small infarcts, perivascular spaces, presumed vascular lacunae, white matter hyperintensities, cerebral microbleeds, and brain atrophy. Patients with CSVD may present with an acute onset, such as lacunar infarction and hemorrhagic stroke, but most have an insidious onset, slowly developing cognitive decline, gait abnormalities, emotional and defecation dysfunction, etc. CSVD is one of the most common causes of cognitive impairment. 18% - 20% of CSVD patients will experience cognitive decline within 5 years, and 45% of dementia patients are caused by cerebral small vessel disease. Domestic studies have shown that the incidence of cognitive and motor function impairment in CSVD is approximately 35.38%, manifested as cognitive impairment characterized by decreased executive function and attention, and motor function impairment characterized by gait disorders, which seriously affect patients' daily lives and may even lead to disability or death. Currently, there is no specific treatment drug for CSVD in clinical practice. The potentially effective treatment measures recommended by the guidelines mainly focus on controlling vascular risk factors, such as blood pressure control, antiplatelet therapy, and statin lipid-lowering, but the level of clinical evidence is low and controversial. Overall, there is a great demand for drugs to treat cerebral small vessel disease in China.

Zishen Yizhi Pills is an effective empirical prescription for the treatment of dementia, which was formulated under the guidance of the academic thoughts of the national master of traditional Chinese medicine, Liu Zuyi, and has been clinically verified for many years. Previous clinical studies by the research group have found that Zishen Yizhi Pills can significantly improve the scores of MMSE, ADL, NHISS, and TCM syndrome scores in patients with VD, effectively improving cognitive impairment and enhancing the quality of life.

Based on the above preliminary studies, we plan to use a multi-center, observational, prospective, non-randomized controlled study design to further evaluate the high-level evidence-based medical evidence of Zishen Yizhi Pills in the treatment of cognitive impairment caused by cerebral small vessel disease (Yin Deficiency and Blood Stasis Syndrome). Research objective: To evaluate the efficacy and safety of Zishen Yizhi Pills in the treatment of cognitive impairment caused by cerebral small vessel disease

(Yin Deficiency and Blood Stasis Syndrome).

# II. Who Should Not Participate in the Study

1. Patients with other neurological diseases causing cognitive impairment, such as Alzheimer's disease, frontotemporal dementia, Lewy body dementia, Parkinson's disease, multiple sclerosis, brain trauma, autoimmune diseases, and genetic diseases;
2. Patients with impaired consciousness, unstable vital signs such as blood pressure, respiration, and heart rate, or life-threatening serious diseases such as severe heart, liver, kidney, and endocrine diseases;
3. Patients who cannot understand and/or comply with the study procedures and/or follow-up due to mental illness, cognitive or emotional disorders, severe visual or auditory impairment, etc.;
4. Patients with severe liver and kidney dysfunction (ALT > 2 times the upper limit of normal or AST > 2 times the upper limit of normal; Cr > 1.5 times the upper limit of normal).
5. Patients who have received traditional Chinese medicine treatment for nourishing the kidney and activating blood circulation within two weeks before enrollment. (7) Patients allergic to the ingredients of Zishen Yizhi Pills.

**3. What do you need to do if you participate in the research**

1. Before you are enrolled in the study, the doctor will ask and record your medical history, and evaluate the patient's condition. If the inclusion criteria are met and you volunteer to participate in the study, an informed consent form will be signed. Your unwillingness to participate in the study will not cause prejudice against you or affect your medical care.

2. If you volunteer to participate in the study, the following steps will be followed:

If you meet the eligibility for inclusion in this study, you may be assigned to one of the following two treatment groups:

1. Experimental group: ① Zishen Yizhi Pill treatment, twice a day, the course of treatment is 12 weeks; ② Standardized routine treatment based on the Guidelines (risk factor control, antiplatelet therapy, rehabilitation therapy, etc.).
2. Control group: standardized conventional treatment (risk factor control, antiplatelet therapy, rehabilitation therapy, etc.) based on the Guidelines only.

Standardized Western Medicine Treatment Based on Guidelines: Referring to the Expert Consensus of Integrated Traditional Chinese and Western Medicine Diagnosis and Treatment of Cerebral Small Vessel

(2024) "," Chinese Guidelines for the Diagnosis and Treatment of Vascular Cognitive Impairment (2024) ", and" Chinese Guidelines for the Diagnosis and Treatment of Cognitive Dysfunction Related to Cerebral Small Vessel Disease (2019) "mainly include the following aspects: risk factor control: blood pressure control, lipid regulation Therapy, blood sugar control, smoking cessation, etc.;

Antiplatelet therapy: aspirin or clopidogrel; Rehabilitation therapy. Course of treatment: 12 weeks of treatment

The study process is divided into screening period (5 days before enrollment), baseline period (day 1 of enrollment), and treatment period

(Week 4 ± 3 days, Week 8 ± 3 days, Week 12 ± 3 days of enrollment), from baseline to visit, we will use the Montreal Cognitive Assessment Scale (MoCA), Mini Mental State Examination Scale (MMSE), Wired Test (TMT-A), Wired Test (TMT-B), Boston Named Test 2nd (BNT-2), Hopkins Language Learning Test (HVLT), Clock Drawing Test (CDT), Instrumental Ability for Daily Living Scale (IADL), European Five-Dimensional Health Scale (EQ-5D-5L), Neuropsychiatric Questionnaire

(NPI), Hospital Anxiety and Depression Scale (HADS), TCM syndrome element score, etc. to evaluate the treatment effect. In addition, we will observe and record adverse events at any time. During the entire clinical research process, we will pay close attention to your condition and any changes, and make corresponding treatment and help in time.

3. Other matters requiring your cooperation

During the conduct of the research project, it is possible that new information about the research methodology will emerge. If new information emerges, your study doctor will promptly inform you and discuss with you whether you are willing to continue participating in this study. If you decide to continue participating in the study, you may be asked to sign a new informed consent form. During the follow-up stage, the doctor may learn about your situation through phone calls, outpatient follow-up, etc.

**4. Possible benefits**

You may benefit from participating in this study: receive standardized medical treatment based on guidelines, as well as Zishen Yizhi Pill treatment (experimental group), and receive regular guidance on secondary prevention and rehabilitation programs, so as to promote the recovery of your limb function and improve your quality of life. You will receive close attention and individualized guidance from the study doctor on your medical condition. You may also not receive direct medical benefits during the study, but the information gathered during the study will help the study physician and researcher to improve their understanding of the study methodology and the treatment of cognitive impairment in cerebrovascular disease, and this information may benefit you and other patients with cognitive impairment in cerebrovascular disease.

**5. Possible risks**

All therapeutic drugs have the potential for side effects. Although no Zishen Yizhi Pill has been found so far

If you experience obvious adverse reactions or any discomfort during the study, such as unexplained rashes, erythema, itching, dizziness, nausea, chest tightness, shortness of breath, pale complexion, cold limbs, difficulty breathing, or new changes in your condition, or any unexpected situation, whether related to the drug or not, you should promptly inform your doctor. He/She will make a judgment and provide medical treatment.

The doctor and the sponsor will do their best to prevent and treat any harm that may result from this study. If an adverse event occurs during the clinical trial, the Medical Expert Committee will determine whether it is related to the test drug. The sponsor will cover the treatment costs and provide corresponding economic compensation for the damage related to the trial, which is stipulated in the "Good Clinical Practice for Drug Trials" in our country.

During the study period, you need to visit the hospital for follow - up on time and undergo some physical and chemical examinations, which may cause you trouble or inconvenience.

**VI. Emergency Measures**

During the study, if you experience an adverse event or reaction, your research doctor will take timely and effective treatment measures to ensure your safety, record it, and decide whether to terminate the trial. In case of a serious adverse event, you should withdraw from the clinical trial, and appropriate treatment measures will be taken immediately.

**VII. Costs Related to Participating in the Trial**

In this study, the treatment cost of Zishen Yizhi Pills will be provided free of charge to the subjects by the Hunan Academy of Traditional Chinese Medicine Affiliated Hospital, the responsible unit of the project. The efficacy evaluation and all scale assessments during the study will also be free. Other related costs will be charged according to the normal hospital charging standards.

**VIII. Confidentiality Principle**

All information about you, including your identity, medical history, condition, physical examination, and laboratory test results, will be strictly confidential within the scope permitted by law. Researchers, quality monitors, and the Ethics Committee are allowed to access your medical records related to this study to verify the authenticity and accuracy of the data collected in this study, but your personal detailed information will not be involved. Your name will not appear in any public materials or reports related to this study.

**IX. Right to Withdraw from the Study**

Your participation in this study is completely voluntary. We hope you can complete the study. You have the right to withdraw from this study at any stage of the clinical

study

for any reason. If you decide to withdraw, please contact your research doctor and answer the questions required for the last visit so that the doctor can accurately understand your situation at that time. Withdrawing from the study will not affect your relationship with the doctor. The doctor will provide reasonable treatment for you based on your condition and the principle of putting patients first. Your research doctor may terminate this study without your consent in the following situations:

1. For the consideration of your treatment;

2. You, as a subject, fail to follow the relevant regulations of the study, such as not having the follow - up on time;

3. The study is aborted.

**X. What Should You Do Now?**

Whether to participate in this study is entirely up to you. You can discuss it with your family members or friends before making a decision.

Before you decide to participate in the study, please ask your doctor as many relevant questions as possible until you fully understand this study.

Thank you for reading the above materials. If you decide to participate in this study, please inform your doctor or the research assistant, and he/she will arrange everything related to the study for you. Please keep this information.

**Informed Consent Form - Consent Signature Page**

**Name of the clinical research project: A real - world study of Zishen Yizhi Pills in the treatment of cognitive impairment in cerebral small vessel disease (syndrome of yin deficiency and blood stasis)**

**Project - undertaking unit: Hunan Integrated Traditional Chinese and Western Medicine Hospital (Affiliated Hospital of Hunan Academy of Traditional Chinese Medicine) Consent Statement**

I have read the above introduction about this study and had the opportunity to discuss this study with the doctor and ask questions. All my questions have been satisfactorily answered.

I am aware of the possible risks and benefits of participating in this study. I know that participation in the study is voluntary. I confirm that I have had sufficient time to consider this, and I understand that:

- I can consult the doctor for more information at any time.
- I can withdraw from this study at any time without being discriminated against or retaliated against, and my medical treatment and rights will not be affected.

I also understand that if I withdraw from the study midway, especially if I withdraw due to blood - letting therapy, it will be very beneficial to the entire study if I tell the doctor about the changes in my condition and complete the corresponding physical and laboratory examinations.

If I need to receive any other treatment due to changes in my condition, I will seek the doctor's advice in advance or tell the doctor truthfully afterwards.

I agree that the regulatory authority, the ethics committee, or the sponsor's representative can access my research data. I will receive a signed and dated copy of the informed consent form.

Finally, I decide to consent to participate in this study and promise to follow the doctor's advice as much as possible.

Patient's signature: Date: Year Month Day

(Or legal representative (signature): Relationship with the patient ) Contact number:

**Declaration by the researcher**

I declare that I have explained in detail to the above participants the content, procedures, possible risk factors, and benefits of this study. I have also provided sufficient answers to the questions raised by the patients. The patients have received satisfactory responses and indicated their understanding. At the same time, I have obtained a signed and dated copy of the informed consent form.

Research doctor (signature): Date: Year Month Day Contact phone number:
